# The Scaleup Costs, Size, and Statistics Challenge to Democratic Deliberation on Health Policy

**DOI:** 10.1111/bioe.70191

**Published:** 2026-10-14

**Authors:** Nir Eyal

**Affiliations:** ^1^ Global Business School for Health University College London London UK; ^2^ Rutgers School of Public Health, Institute for Health, and Philosophy New Brunswick New Jersey USA

## Abstract

Current fashion supports aligning health policy with the recommendations of deliberative forums, or mini‐publics. These forums' participants are selected randomly from the general public (sometimes with overrepresentation of direct stakeholders or of underrepresented sectors). After being informed and exchanging public reasons with fellow participants under discursive rules that engage all as equals, participants vote on and sometimes rank policy recommendations. According to supporters, a deliberative democratic process that holds such forums and translates their recommendations to policy legitimizes resulting policy. That's thanks to this process's tendencies to strengthen democratic consent, to uncover which policy option is objectively best, to align policy with lay normative outlooks, and to educate the public. I argue that one tri‐partite feature of nearly all instances of democratic deliberation on regional, national, and global health policy under current best practice, which I call the “Scaleup costs, Size, and Statistics” (“3S”) challenge, largely blocks these alleged welcome tendencies. In particular, barring radical changes, true democratic deliberation is expensive to scale, keeping feasible mini‐publics small and irregular. That makes concluding from mini‐public recommendations about the population's hypothetical consent, values, and will statistically unreliable. It also caps low mini‐publics' contribution to public education. In these ways and in others, the long list of alleged welcome tendencies of proper democratic deliberation on health policy, and the related contribution to political legitimacy, is largely a fiction. Public deliberation does have modest true merit, for example, providing relevant qualitative input about the lay public's occasional factual insights and many endemic errors; and making the public feel “listened to,” whether or not it is. But those merits are modest, contingent, and most accentuated under different institutions than envisaged in deliberative democratic literature. Certainly there is room for democratic deliberation, potentially of new breeds, but only as part of the mosaic of health policy design, alongside familiar methods like general elections, public surveys, representative government, and expert decisionmaking. The main question is which method to use when.

## 1 Democratic Deliberation on Health Policy

Democratic deliberation has captured the imaginations of political philosophers since the 1980s. On health policy, it has been put to practice since the 1990s. Citizens' juries and larger citizens' assemblies, parliaments, and conventions have explored topics ranging from healthcare allocation and financing through abortion to gene editing [1, e.g., pp. 13–14]. By now, several global citizens' parliaments, for example on gene drives and on global warming, have also taken place. An unfolding political philosophy debate about “lottocracy”, in which randomly‐selected citizen teams are tasked to deliberate and set (nearly) all policies instead of elected officials and professionals [1, 2, 3], sometimes presents lottocracy as a radical form of deliberative democracy [1, 2, e.g., ch. 11].

In *Democratic deliberation,* policy is first discussed with ordinary citizens. They receive relevant information and then exchange public reasons for competing policy options on equal footing with one another. Their reasons and post‐deliberation opinions inform resulting policy.^1^

Recently, *best practice in democratic deliberation* (namely, its proper not actual instantiation), on either health policy or in general, is also associated with:

a. *Broad inclusion*: Deliberation must engage ordinary citizens, especially disenfranchised and underrepresented sectors. Deliberations exclusively among aristocratic rulers and socioeconomic elites, elected representatives, or ivory tower experts are not deliberative *democratic* [1, 17, 18, 19, 20].^2^
b. *Random participant selection:* Invited delegates must be chosen randomly and form a cross‐section of society [1, 2, 21], potentially with overrepresentation of or added weight to underrepresented minorities or the greatest stakeholders (say, the relevant patients).
c. *Balanced information:* Deliberation must only begin after delegates learnt basic facts on the policy dilemma, for example, from an authority or from debating experts [1, 22].
d. *Dialogue and an open mind:* Thorough deliberation must give all a real opportunity to reconsider convictions. It goes beyond merely inviting topics or quick feedback for what otherwise remain deliberations among elites.^3^
e. *Tendency of deliberative decisions to translate into later policy:* Deliberative forums' roles are not merely consultative, to provide input for elite decisionmakers who remain final arbiters and may altogether ignore the advice [1, 13, 23].^4^ Increasingly, (ranked) recommendations based on post‐deliberation votes and potential^5^ consensus statements are expected to be at least somewhat directive. Even when mini‐public recommendations do not set policy, policymakers are ideally expected to be accountable for impementing or closely considering them [e.g., 24].

This article's scope is democratic deliberation on regional, national, and international health policy under these best practices and in current environments. That scope means we set aside (i) real‐world democratic deliberation, whose problems might be irrelevant to best practice; (ii) non‐inclusive deliberation, e.g. ivory tower conversations; (iii) democratic deliberation on topics outside health policy; (iv) local mini‐publics like community forums and townhall meetings; *and* (v) mini‐publics in very different environments than current ones, where deliberation is mandatory, the public employs no politicians and professional experts, the populace is on the scale of Ancient Athens, or other radical differences obtain.

Some bioethicists see democratic deliberation on regional, national, or international health policy as imperative for controversial decisions, not optional [28]. In medical and scientific research grants, policy, and regulation, components of such democratic deliberation are increasingly mandated (see the introduction to this symposium). In this spirit a 2025 high‐level public meeting of the Council of Europe “hosted an exchange of views on”Deliberative democracy—from “nice‐to‐have” to essential for thriving democracies” [29].

Philosophers may be expressing a similar sentiment when they write that proper democratic deliberation is key to the “legitimacy” of policies [e.g., 18, 30], especially health policies [e.g., 12, 13, 22, 31, 32, 33, 34]. If democratic deliberation is always neccessary for or at least substantially “strengthens” [23] legitimacy then it always matters greatly.^6^ While “legitimacy” carries many meanings in contemporary political philosophy [35], it is often deemed a precondition for permissible or authoritative political action, lexically more urgent than justice [compare 36], itself lexically prior to, for example, maximizing utility and maintaining a *modus vivendi*.

What might make proper democratic deliberation an imperative for health policy, especially the stable fundamental imperative that political legitimacy is taken to be? The literature offers many justifications, such as better representing the will of the people and manifesting impartiality. This article argues that virtually all extant justifications for proper democratic deliberation on health policy in current environments, and especially justifications purporting to link such deliberation to legitimacy, are largely blocked by three interrelated descriptive features of democratic deliberation. Section II presents what I call the scaleup costs, size, and statistics (3S) challenge to democratic deliberation on health policy. It shows that democratic deliberation is usually expensive to scale (“scaleup costs”); that therefore, to be feasible, deliberative democratic forums must remain small and irregular (“size”); and that all that makes most related quantitative conclusions on the general public's hypothetical consent, values, and will statistically unreliable (“statistics”). Section III then explains how 3S renders the long list of welcome tendencies typically ascribed to democratic deliberation largely a fiction in the case of best‐practiced democratic deliberation on nonlocal health policy in current environments. A conclusion assesses what remains of the case for democratic deliberation on health policy.

## 2 The Scaleup Costs, Size, and Statistics (3S) Challenge to Democratic Deliberation on Health Policy

Imagine a US health policy poll whose respondents are representative of the US population in terms of their gender, race, ethnicity, religion, sexual orientation, conservative/liberal self‐definition, and party affiliation breakdown. There is something unusual about that survey, though. The number of participants is two (2).

What should we make of that “survey”?

In a sense, a two‐participant survey can be representative of the US population breakdown. It could have precisely the number of women, whites, liberals, Native Alaskans, Oklahomans, gays, Republicans, and so forth as their share in the general population, rounded down or up to 0, 1, or 2—whichever is closest. It is challenging, but doable, to keep the two respondents representative on all these fronts. The two participants can be selected randomly from a pool of suitably stratified Americans.

But that survey is worthless, and “representative” only superficially. It generates far too much random noise to be informative. It is utterly unreliable. It could easily suggest public trends that are barely there, or miss big trends.

This section argues that high cost at scale will keep the bulk of feasible democratic deliberation on regional, national, and international health policy too small and irregular for ample statistical significance and power to reflect the relevant publics' positions, values, and will. Mini‐publics with 20 or even 200 delegates are better than 2‐repspondent “surveys” only to a degree, not categorically. This is the scaleup costs, size, and statistics (3S) challenge to basing health policy on mini‐public recommendations as binding on health policy.

### 2.1 Scaleup Costs

Start with the cost at scale. Supporters of democratic deliberation concede that most mini‐publics cost plenty per participant. For meaningful deliberation, an ordinary citizen needs to be covered for travel, lodging, and food during deliberations. Recent recommendations, mindful of inclusivity (element “a” of current best practice) are also to “provide… stipends and childcare, so all can participate,” all of which cost dearly [37].^7^

I was unable to find a systematic review of the total costs of democratic deliberation. But case discussions on *Participedia*, a crowdsourcing platform on public participation, cite widely varying costs that run as high as $17 m [39]. France's Citizens' Climate Convention cost upwards of €6 m [1]. Deliberative Polling® publications and related website [40] seem as eager to share price tags upfront as expensive clothes boutiques are. But a central British pro‐deliberation organization calls the costs of Deliberative Polling “high”, owing primarily to “participant recruitment and expenses” and media involvement [41].^8^

James Fishkin, who trademarked Deliberative Polling®, responds that “The whole process … can also be conducted entirely online, with video‐based discussions, to make it cost effective” (Fishkin 2021, S23). He has co‐conducted multiple online‐only deliberative polls. However, it remains unclear how open minded (element “d”) online deliberation can be [compare 42]. Corporations have already discovered that some of the meaningful, open‐minded deliberations that *they* need cannot happen online but in person only. By 2024, 70% of companies have instituted formal policies requiring some in‐office time, which 93% of their leaders strongly support (Teter 2025). There is evidence that in‐person meetings generate more ideas, increase understanding of (crucial) nonverbal cues, build trust, and reduce the temptation to multi‐task, in comparison with online group meetings (Ghoshal 2023; Akhter 2024).

Additionally, other supporters of democratic deliberation are less hopeful about online cost cuts when deliberations are kept inclusive (element “a”):

Some argue that online deliberation is less expensive than in‐person processes, but the costs are similar when designed to be as democratic as possible. The new wave pays much more attention to inclusivity. For many online citizens' assemblies this year…, participants without equipment were given computers or smartphones, along with training and support to use them… [37].

Admittedly, “deliberation” in the minimal sense of letting lay publics post online notes which may or may not be read and discussed concerning professionals' draft decisions remains cheap. However, truly inclusive (element “a”) and truly dialogical (element “d”) democratic deliberation seems to remain costly per participant.^9^

### 2.2 Size

High cost at scale forces deliberation on health policy to engage relatively few participants, occasionally and irregularly. Kyungdo Lee's contribution to this symposium criticizes South Korean health policy deliberative workshops with 30 participants each—statistically inadequate for representing a nation of 50 million. Some hope that deliberative assemblies sidestep this challenge: “An assembly, at 100+ people, is much bigger than a jury, typically around 20–25 people. Assemblies can sometimes claim to be more or less statistically representative of some larger population…” [44]. But surveys with 100 participants (as in Ireland's Citizens' Assembly) or 150 participants (as in France's Citizens' Convention on Climate), or even “hundreds” of citizens (as in Deliberative democrat Hélène Landemore's ideal assembly [1]) are statistically inadequate for representing millions. A sample‐size calculator reveals that for a simple Yes/No question, only several thousand respondents might be enough.

Strikingly, central guidance on how to conduct democratic deliberation seems satisfied with such participant numbers, and adds no further requirements on statistical significance and power [e.g., 45; article 3]. Recent statistical preoccupation is with proportionate representation (a form of what philosophers call “descriptive representation”), not with participant numbers. A new defense of democratic deliberation on health financing hails an Israeli citizens’ parliament with “a stratified random sample of 1500 people from the adult population with an over‐sampling of minorities and new‐immigrant populations” [46]. It neglects the actual number of participants, on which the article it cites even changes versions—”132” at one point and “130” at another [47]. Both seem stunningly indifferent to the number of actual deliberators representing millions.^10^ Discussing a pilot citizens' assembly to represent 26 million Australians, even critics concede, “Although only 23 people participated, this number is on the order of magnitude recommended by the citizen deliberation literature” [48].

High per‐participant cost also keeps deliberative forums on health policy short (a few days in total),^11^ irregular, and *ad hoc* [42]. Total deliberation time remains small even across health policy deliberative events—unlike the jury work to which Landemore [1] and others compare it.^12^

### 2.3 Statistics

Excessively small sample size raises two statistical challenges. The main challenge is random noise [50]. Whether simple or deliberative, an excessively small poll is not powered to detect reliably trends, say, a consensus in support of a certain policy, a large minority for it, or a hung public. False negatives are too likely. A small‐size poll also lacks the statistical significance to reliably avoid suggesting trends that are not there. The margins of error are simply too large for reliable determinations. While in a large sample, random noises in different directions might have canceled each other out, that rarely happens in a small sample. And a cross‐section of society on many demographic and attitudinal bases [as e.g., 51, 52 propose] forgives somewhat smaller sample sizes, but not numbers as small as noted.^13^

Several factors exacerbate this statistical challenge. While an overwhelming consensus might have been roughly discernible even with a small sample size, supporters recommend democratic deliberation precisely for those questions on which there is “reasonable disagreement”, understood as *no* clear consensus [31, 32].^14^ They also recommend democratic deliberation for health policy—where “the winners and losers win and lose something very important, namely health, or even life” [13, 22], so statistical standards should be exacting.^15^ Finally, participation in deliberate forums is burdensome, so the difference between the number of participants and those invited to participate is typically large [1, p. 105], increasing random and systematic noise.

Admittedly, on the rare occasions that we are only interested in whether there are *any* supporters for a certain view, or uninterested in what the public supports only in changes to what it supports following deliberation (the official^16^ focus of Deliberative Polling), the sample size of many deliberative forums is adequate. And rarely, the general limitations I mentioned are absent.^17^ On the other hand, the sample size is especially inadequate when the outcomes sought are multi‐option (e.g., Which of policy options A, B, and C is best?); and when what is sought is correlation between a certain answer and a certain demographic affiliation (e.g., “Policy A was rejected by Christians”).

A second statistical challenge when sample size is inadequate is sampling bias. This challenge arises even on the assumption that statistical representation is exclusively a matter of descriptive representation. On some questions, the social divisions that best predict a respondent's position are combinations of multiple demographic and/or attitudinal characteristics: suburban white females with ≥ 1 children might have very different opinions and voting patterns than those of suburbanites in general, of white females in general, and of parents in general. The experiences and opinions of African‐American men in America's northeast may differ greatly from those of African–American men in the south, and in ways that do not resemble other US north/south differences.^18^ Some highly predictive categories (such as how many decades the respondent has lived) admit of important subcategories. With relatively few delegates, it is hard to represent proportionately each and every one of the small and combinatory predictive sectors. Invite white females to participate, and those who accept might be urban or childless. The partial solution of weighting higher the votes of any suburban white females with > 1 children is unavailable when none are surveyed—as will frequently occur in a small cohort. Unfortunately, “if a [deliberative] jury is unrepresentative to begin with, in either its initial attitudes or its demographics, that starting point could distort the eventual decision” [52].^19^

Admittedly, citizens' parliaments and assemblies are no worse in this respect than actual parliaments and the UN general assembly. For some deliberative democrats, therefore, instead of “a perfect mirror of the characteristics of the world's population, … Much more meaningful is comparison with the other forms of representation currently available in the global system” [44]. However, when fairly few representatives are elected, each will have been scrutinized by voters for likelihood to promote their particular agendas (a so‐called “selection” mechanism of accountability [56]), and each knows that satisfying many voters would facilitate re‐election (a “sanctions” mechanism). Accountability through elections is unavailable when invited delegates are chosen randomly [3, ch. 4], as they are in best‐practiced deliberative forums (element “b”).^20^

Some supporters dismiss the documented problems in the practice of real‐world democratic deliberation as irrelevant to the fundamental ideal [26, 58], claiming that those are resolvable by tweaking deliberative practices [e.g., 59]. When participant numbers are statistically inadequate, they might answer, rather than doing away with democratic deliberation altogether, we should simply increase those numbers. Nonetheless, for most health systems, more deliberators would make democratic deliberation entirely unaffordable. In that way, 3S affects not only *actual* democratic deliberation, but all *feasible* forms of proper democratic deliberation on nonlocal health policy in current environments. It is a robust challenge.

How does 3S differ from nearby challenges to democratic deliberation on health policy? Daniel Hausman's contribution to this symposium identifies the *cost* problem that deliberative forums “are too expensive to address large numbers of issues.” By contrast, 3S arises even when a single question is on the deliberative ballot.

3S is not the *size* challenge that “For deliberation to satisfy the qualities that recommend it, i.e. listening, reason‐giving, common ground and adjustment of positions on the basis of better reasons, it must take place in small groups” [60; and see also Hausman's contribution to this symposium]. *That* small size may be achieved by dividing deliberators into small subgroups.

The target of 3S is deliberation about regional, national, or global health policy, not local policy. A townhall can be statistically adequate for a small local population. And sometimes, when the reason that one's position is under‐represented in a townhall is solely one's own avoidable failure to show up, that partly condones the underrepresentation. Mini‐publics on regional, national, or global health policy simply cannot include all stakeholders.

Combined, two innovations may initially seem to mitigate 3S. First, in *structural democratic deliberation*, multiple individually‐small or biased deliberative forums purport to be adequately representative together [61]. What if many (small) deliberative forums were held, and their resolutions were meta‐analyzed into conclusions about the public's hypothetical post‐deliberation opinions on multiple other topics?

Such meta‐analyses would be highly speculative. A person's post‐deliberation opinions on gene therapy, for example, hardly reveal her opinions on health sector financing. Speculating would invite both real and merely‐suspected manipulation, exacerbating instead of allaying public distrust [compare 52].

A second innovation may manifestly block such manipulation, and save trust. What if large mini‐publics engaged a large single group of randomly‐selected lay delegates on many questions, and each delegate's post‐deliberation choices served to train an *artificial* personal “bot” for him or her, with open, impartial protocols? Just as a bot quite reliably represents philosopher Peter Singer's views on old and new questions based on his large corpus [62], these personal bots could represent these delegates later. Each delegate's bot would then deliberate and choose reliably in his or her name on any of a great many other questions. That might be thought to reliably mirror that delegate's hypothetical deliberations and votes on any of a great many policy questions. And together, these bots could be thought to represent the public's post‐deliberation choices on very many policies, absent further expensive forums. The substantial one‐time investment of running many large mini‐publics with the large group of delegates and of training bots on the combined results would pay for itself.

However, it is already hard to recruit delegates for a single large mini‐public. To recruit many to as many mini‐publics as necessary in order to train bots (and statistically “drown” the situational influences that presumably affect lay persons more than Peter Singer) seems infeasible. Moreover, participants who volunteer to so many forums would tend to have lots of spare time, extreme need of the incentives on offer, strong desire to influence healthcare, or still other distinctions—a violation of true random selection (“b”).

## 3 Questioning Standard Defenses of Democratic Deliberation on Health Policy

Why does 3S matter? Let me trace how 3S largely foils standard justifications of democratic deliberation, on nonlocal health policy under its best practice, barring radical changes.

### 3.1 Epistemology

It is now common to ground democratic deliberation in its alleged epistemic advantage over alternatives like expert decisionmaking [59].^21^ First, both getting informed and deliberating (parts of deliberative best practice) tend to reveal what choices are objectively best [59]. Second, the lay public, especially silenced groups, can have fresh perspectives that experts with uniform training and backgrounds and potentially tunnel vision and dogma can miss [1, 2, 59]. Third, the sheer inclusion in deliberation and decisionmaking of more people than just a handful of experts or representatives already increases substantially the chance of selecting the best policy so long as, post‐deliberation, each person has > 0.5 chance of selecting the best one [1, 63].

The first of these three alleged advantage is irrelevant and the other two are undermined by 3S. The irrelevance is because democratic deliberation and expert deliberation alike involve information and deliberation. The merits of being informed and of deliberating need not make democratic deliberation preferable to expert decision‐making.

Moving to the second alleged epistemic advantage, it is true that stakeholders on the ground tend to understand implementation challenges better than so‐called professional “experts” and political representatives [64]. And many professional experts are bound by their published dogmas or captured by special interests [2]. Surely, however, professional experts retain some distinctive strengths. They passed educational and professional filters. They have years of training and of experience considering similar policy dilemmas. It is admittedly an overgeneralization that “the vast size and complexity of modern government make it unlikely that most citizens can ever reach the levels of knowledge and rationality required by deliberative democracy, even if they were better informed than they are at present” [65].^22^ However, whose deliberations are likelier to discover the truth is partly an empirical question that political philosophers alone are unqualified to answer. As with other social science questions, the answer is likely to vary. For *some* pertinent aspects of policy, *sometimes*, most deliberators selected randomly from a cross‐section of society are presumably far less capable of deliberating effectively than professional area experts, even after information sessions (element “c”), which per 3S must remain brief.

Landemore concedes that individual experts are sometimes likelier to decide right than individual lay deliberators, but insists on the greater collective wisdom of democratic deliberators. Her main ground is that even if together, experts are likelier to reach higher local optima, inclusive citizens' assemblies tend to be better at identifying higher non‐local optima, and are hence stronger at resolving the complex problems of politics. She repeatedly cites the “Diversity Trumps Ability” theorem [66] and concludes that inclusiveness is “ecologically rational”. In particular, Landemore argues,

a. “More inclusive assemblies are simply more likely to be cognitively diverse,”
b. Cognitively diverse groups move more nimbly to higher non‐local optima than cognitively uniform groups, *and*
c. The advantage of reaching non‐local optima matters enough that, given (a) and (b), inclusive citizens' assemblies are overall stronger than experts at resolving the complex problems of politics [1, 59].

The 3S challenge questions each of these assumptions. Start with (a), Landemore's exceptionless association of inclusivity with greater cognitive diversity. My personal impression is that early on in our learning curves and paths to self‐confident knowledge, many of us rigidly swear by common generalizations and other opinions that had been strongly aired to us. Only later do we realize that these opinions admit of limitations and exceptions. Per 3S, mini‐publics' active deliberation must remain brief. That curtails how much lay delegates, comparatively new to the topic being discussed, so comparatively early on their learning curves, can gain ample confidence for true open mindedness during mini‐publics. This might be why, on hard questions, most democratic deliberators conform to strong opinions aired early in deliberations [67]. By contrast, academic experts normally undergo area training, then practice for years in the area, questioning both rules and one another's judgements—something that good academic institutions incentivize. The similar training and—admittedly often privileged—shared backgrounds of experts oil interactions and can keep expert groups' laser focus on sharpening then tackling relevant disagreements. The upshot, partly on account of 3S, can be greater *relevant* cognitive diversity among demographically uniform expert groups than among demographically inclusive groups whose descriptive representativeness is greater. Iris Marion Young was admittedly right to find some fault with “deliberation among gentlemen who already share basic understandings” [17]. But such deliberation can with relative comfort broach disagreements, enhancing viewpoint diversity. On social science, exceptionless generalizations, including Landemore's (a), usually fall flat.^23^

I agree with Landemore's (b). As Dan Hutton Ferris and Johannes Kniess' contribution to this symposium puts it, non‐expert opinions are often “wildcards”—usually off but occasionally true gems that reach higher non‐local optima. Accordingly, a qualitative approach to deliberation can improve expert decisionmaking by documenting *potential* gems for careful expert screening. Unfortunately, quantitative post‐deliberation polling (element “e” of current best deliberative practice) conceals those rare gems. As 3S contends, active deliberation is too brief for unusual gems to gain widespread support on time. And the vote that follows tosses out rare gems as small minority views. Ironically, then, philosophical supporters of deliberative democracy are the ones blocking its real potential to generate interesting ideas through qualitative inquiry by demanding instead the legitimacy that they associate with post‐deliberation majority voting. Indeed, kicking off mini‐publics with information sessions (element “c”) can also block some of the important potential qualitative input, for example, early warnings about common false beliefs (see Robert Steel's contribution to this symposium). The information session lowers false belief rates among deliberators, masking some of the urban myths that it would have been useful to expose early. The general tension between informing people and collecting interesting qualitative evidence from them is why ethnographers and qualitative researchers do *not* start interviews by using the “non‐forceful force of argument” to assimilate informants' worldviews. That would have preempted important input.^24^

Moving to Landemore's (c), even when better at non‐local optima, cognitive diversity is sometimes on balance an epistemic disadvantage. Imagine a five‐member committee to select the best analytical metaphysics essay prize. Which committee composition is epistemically more promising: exclusively analytical philosophers with somewhat uniform training in Anglo‐Saxon academic institutions, or a diverse committee consisting of four such analytical philosophers and one continental philosopher, anthropologist, or nonacademic who has never before meditated metaphysically? The cognitively more uniform group is usually more promising. It cumulatively has a little greater area expertise: 5 > 4. But ts main advantage is that it need not waste precious time explaining or arguing over basics, and could devote longer to debating which essay is most innovative, convincing, and the like. If so, cognitive uniformity is *sometimes* an epistemological advantage.^25^

A final argument for the collective wisdom of democratic deliberation is based on the Condorcet Theorem. A large number of decisionmakers, each with an independent probability above that of a coin flip to get things right, voting sincerely, are under common decision processes very likely to get things right—more than a few decisionmakers with higher personal probability of getting things right. Per 3S, however, in democratic deliberation on policy, the number of delegates is small.^26^

The verdict on the epistemologically‐best system overall is complex and partly empirical. Per some summaries of the evidence, democratic deliberation can “lead to worse decisions than would have occurred if no deliberation had taken place” [67]. 3S strengthens that tendency.

### 3.2 (Re‐) Consent

Simple majoritarian consent may be insufficient to legitimize controversial health policy on matters of life and death. Certainly in many countries, majoritarian processes are flawed or altogether absent. When they are democratic deliberation can provide the necessary added legitimacy or authority, holds another defense. By analogy, a volunteer army service may be legitimate for many purposes, but for a high‐risk mission, enhanced individual consent can be necessary.^27^ When it comes to public policy, democratic deliberation is the closest analogue to enhanced individual (re‐)consent, the argument continues. So democratic deliberation can be necessary for health policy justification (or even conclusive: once the people's forum has “spoken”, ivory tower experts and elected representatives must abide, say others [13, 22, 34]).

Suppose that, in line with this defense, the post‐deliberation consent of a majority of the public would have legitimized otherwise‐illegitimate policies. Per 3S, the number of actual delegates in most deliberative forums would remain too small to indicate reliably what the majority of the general public would consent to post‐deliberation, and certainly would not constitute its actual consent. After 11 months of deliberation, British Columbia's deliberative citizens' assembly recommended a new electoral system, only to be promptly rejected in the general referendum that followed.

Perhaps when ordinary majority rule makes health policy decisions near‐legitimate, even the imperfect deliberative consent that 3S permits seals their legitimacy? But if as 3S contends mini‐publics provide statistically unreliable evidence of consent, their added democratic value is only modest. So the suggestion is odd. Take individual patient decisions as an analogy. When a risky and important personal medical choice, say, to donate an organ to a stranger, is necessary, it would be odd to authorize that choice on the added strength of poor‐quality consent, say, on the patient's toddler's assurance that her parent wholeheartedly consents. One cannot entirely rule out the possibility that while both the patient's original consent and the added poor‐quality consent are individually invalid, their combination would provide just enough evidence of what the patient would want if fully autonomous. But when the quality of the added evidence of consent is small, that is rare—in individual patient decisions and in public decisions on health policy.

The same statistical unreliability questions also Joshua Cohen's [30] famous suggestion that “outcomes are democratically legitimate if and only if they could be the object of a free and reasoned agreement among equals.” Any outcome could occasionally be selected in a free and reasoned agreement among equals. People are different, and for any outcome, some small collection of free individuals who reason as equals would sometimes elect it depends on the luck of the draw.

Of course if the community or its true representatives publicly authorize a mini‐public to speak in their names (“Let us pursue whatever health policy this mini‐public selects”), then that mini‐public would usually have that authority. One reason why even tiny juries enhance verdicts' legitimacy may be an implicit “Let us pursue whatever verdict this jury selects.” But this is not saying much. “Let us pursue whatever policy this uneven‐coin flip selects” would have authorized an uneven‐coin flip. What 3S reveals is that the *intrinsic* qualities of best‐practiced democratic deliberation hardly increase health policy legitimacy.

### 3.3 Promoting Lay Values

Bioethicists' demographics and their opinions on normative health policy diverge from those of the general public [69]. Elected politicians are selected to promote the lay public's values or have incentives to do so [56]. But unelected Bioethicists will often implement their own values not at all the prevailing values. Another defense of democratic deliberation points out discrepancies between (bioethics) experts' and the public's values that admit of reasonable disagreement [13, 22, 34] and calls for inclusive bioethics [69] or inclusive deliberations on health policy [12, 13, 22, 23, 34].

Let us set aside the complex normative question whether reasonable disagreement on values calls for promoting the general public's values [57]. Even if it does, 3S makes it statistically questionable that democratic deliberation captures the lay public's values. At best, the recommendations of deliberations with a sliver of the public are typically a mix of those values with enough noise to mar statistical interprepretability.

Fishkin may concede as much about especially small “citizens' juries”, but insists that larger forums are different:

Any very small and certainly unrepresentative collection of a few citizens in a jury‐like model raises the question, why listen to them about the question of public will? …for a question of public will, I would argue, we need a microcosm of the public. Such a microcosm, if it is truly representative in attitudes and demographics, can speak for the public as a representation of what the public would think, under good conditions for thinking about the issue [52].

However, per 3S, even comparatively large mini‐publics are unreliable signals of public will.

### 3.4 Manifest Impartiality

Professional experts and professional representatives are often partial toward their near and dear. Many also receive campaign contributions, “revolving door” options, or even kickbacks. Even when appropriately impartial and fair‐minded, suspicion of their partiality or corruption breeds social tensions. Better rely on deliberative forums, argues another defense of democratic deliberation [1, 2, 21, 23].

This argument sometimes neglects that lay delegates can also be partial and corrupt by pretending to use public reasons while in fact prioritizing selfish interests or their near and dear. Members of deliberative forums may promise to reason impartially, but need not keep the promise, notwithstanding optimistic statements like “since the members will be required to cite only reasons relevant on all sides, they will also be inhibited from lobbying” [70]. The same grounds that warrant realistic suspicion of representatives and experts are also grounds for similar suspicion of deliberative democratic delegates.

Supporters of democratic deliberation respond that thanks precisely to the typical brevity and ad‐hoc nature of deliberative forums, lay delegates lack the time to get corrupted [1, 2]. However, lay delegates also lack some buffers against corruption common among officials and experts, including oaths of office, a professional ethos, regular anti‐corruption and fair‐mindedness training, salaries that make it affordable to reject bribes, and the risk of losing professional careers. Some lay delegates see their roles as defendants of this or that sector or stakeholder. Whether partiality and corruption are overall more likely for professionals than for lay delegates is thus an open and largely empirical question.

Supporters may also respond that even when a lay public is highly partial, cumulatively its post‐deliberation decisions may roughly capture public interest. When each member of a cross‐section of society prioritizes their near and dear, most everyone's interests get represented by someone. So long as deliberative forums retain ample directive power (element “e”) to thwart corrupt officials' meddling, the more manifestly impartial will policy be. However, 3S undermines this response as well. On health policy's matters of life and death, statistical unreliability will frequently leave officials and professional experts no choice except to overrule post‐deliberation recommendations that seem off. Yet the whole argument assumes that officials and experts are unacceptably partial.

The manifest guarantee of impartiality might be strongest if decisionmaking were delegated to a third party with no horse in the race. A committee of international experts whose near and dear remain unaffected is one option. But it would violate broad inclusivity (“a”).

### 3.5 Fair Outcomes

The distributive outcomes of deliberations with members of a given minority turn out empirically to be more favorable to that minority and hence, when that minority is disadvantaged, comparatively egalitarian or prioritaritarian. In these and in additional ways, best‐practice democratic deliberation on health policy may result in fairer resource allocation than alternative decision methods—another argument in its favor [23, 46, 71, 72].^28^

Empirically, democratic deliberators do not prioritize disadvantaged demographics in general, just those disadvantaged demographics represented in the same forum as these deliberators. And per 3S, these forums are too small to include membership from most small minorities [52]. Moreover, deliberators, often correctly aware that they were randomized from their own demographic, may feel fiduciary obligations to advance its interests, even when advantaged. On balance, therefore, advancing large, advantaged demographic groups at small minorities' expense *may* turn out to be worse under democratic deliberation. Whether it does is an empirical question, not something for philosophers to lecture about.

### 3.6 Equal (Opportunity For) Voice, Deliberation and Decisionmaking

It is typically good for us, perhaps intrinsically, to voice our opinions, get heard, be offered public reasons, deliberate them, and influence final policy decisions, as well as to have the opportunity to do all that [73].^29^ By recruiting people who rarely get to do all that, democratic deliberation equalizes somewhat the distribution of these important non‐fungible goods, another alleged advantage over government by professional experts and representatives. There is beauty when a silenced, shy citizen speaks up and reasons as an equal in mini‐publics [1, 20], which has converted observers into supporters.

Per 3S, however, deliberative forums are small and held irregularly. For each member of the general public, it is rare to take part in their deliberations. Nearly all will never win the random “lottery” and get invited to voice their worldviews, consider responses, and the like. The improvement in how these important goods are distributed is therefore negligible.

In a thin sense of “opportunity”, democratic deliberation still gives even uninvited members of the public an *opportunity* to deliberate and decide. When all have the same chance of being invited to deliberations, that opportunity is equal: “one person, one lottery ticket” [1]. However, (equality in) *that* opportunity is worth little. Take what may initially seem like an unfair analogy. An equal chance of 1:100,000 to land a prestigious job is not an important (equal) opportunity. It improves one's situation in life and its fairness only negligibly. This is why wide economic and professional gaps are barely excused when a strict crystal ceiling is replaced by one with a tiny hole, for a few lucky individuals to enter. Per 3S, the probability of being invited to a mini‐public is small as well—1:100,000 in some of the examples above.^30^

Things are different in truly‐free general elections, to which all citizens are invited to contribute. Results that are disappointing to those who chose not to vote stem partly from their own choices not to. That keeps these results fairer from some desertarian and luck‐egalitarian viewpoints.

### 3.7 A Good Way out of Collective‐Decision Impasses

Democratic deliberation is also praised for its handling of several complications to selecting a policy, e.g., under initial indeterminacy. These complications range from reasonable disagreement on which policy would be more just [74] through intransitive collective preferences [34, 75] and other preferences that are neither indifferent between nor stronger for either policy considered [22, 76] to other political impasses [77]. First, democratic deliberation can alter individual preferences and preempt or resolve the complications, for example, by turning multi‐peaked collective preferences single‐peaked [75, 77, 78]. Second, talking together through these complications is said to treat everyone with respect, impartiality, and procedural fairness. In these two channels, democratic deliberation is thought to bolster normative legitimacy [12, 23].

Let us look at each channel in turn. Preempting or resolving complications is surely welcome. One wonders however if alternatives to proper democratic deliberation would not resolve them even faster. Might privately fleshing out potential compromises with one's legal counsel reach compromises faster? Might letting everyone's lawyers or political representatives iron out differences without any deliberation between lay stakeholders work faster still? What about precommitting to accept compromises proposed by a demonstrably impartial artificial intelligence? Be that as it may, the 3S challenge to any post‐deliberation recommendations (especially when those are ranked) may mean that deliberative forums lack statistical standing to tell which compromises the full population recommends.

Might democratic deliberation bestow legitimacy even when it fails to preempt or fully resolve these complications, thanks to its procedural fairness? It may seem obvious that when the government abides by the verdicts of deliberative forums, everyone's normative outlooks and hence their moral powers are taken into consideration and treated respectfully. But 3S makes it impossible to conclude with confidence that post‐deliberation polling captures everyone's normative outlooks. Admittedly, there may remain social value in giving the impression that everyone's normative outlooks have been considered. As the next subsection elaborates, feeling heard can boost people's social cooperation, whether or not they are truly heard. Far from paradigmatically respectful, however, ramping up social cooperation by misleading people into feeling heard when they are not is somewhat *dis*respectful. As I elaborate later, 3S undermines democratic deliberation's purport to achieve legitimacy through more perfectly respectful processes.

### 3.8 More Trust, Cooperation, and Public Spiritedness, Less Polarization and Populism

Distrust in government and in the healthcare enterprise is gnawing through Western democracies, undermining public health compliance and cooperative politics. A recent argument for democratic deliberation is that it would help restore trust and cooperation and reduce tensions, polarization, and populist voting [1, e.g., pp. 27ff., 18, 29].Working together to identify the best joint health policy could nurture comradery and the understanding that policy is complex and that even disappointing decisions were legitimate [13]. Empirically, contribution to such deliberations tends to instill public spiritedness and a greater understanding for the complexity of the decisions facing health officials [47]. It reduces both support for populist agendas [18] and, when attitudinally diverse, polarisation [78]. It may coopt participants to implement willingly the policy jointly selected [23].^31^ And while such effects might be dismissed as manipulation [64], democratic deliberation could be said to achieve them without manipulation, simply by preempting speculation about rigged decisionmaking, revealing the health system's genuine trustworthiness, heeding citizens' own values and interests, and respectfully reasoning with them. Just as jury discussions are since Alexis de Tocqueville often said to enhance members' civic education [1], other deliberative forums allegedly foster delegates' civic education and knowledge, enhancing both later deliberative forums [79] and political life in general [1, 13, 58, 80, 81].

Per 3S, however, delegates comprise only a tiny portion of regional, national, and global populations. The vast majority will have never participated in mini‐publics or talked to participants, keeping these effects politically marginal. Surveys to discover what portion of publics has even heard of most local, regional, and global citizens' juries and assemblies could be eye‐opening. If few did, that should cast doubt about the promise of democratic deliberation to enhance *population* trust and civic education. Admittedly, when conjoined with substantial media attention, public deliberations can reach and inform a wider public [52]. But media involvement supersizes costs [41], which, on top of 3S's already‐high cost per participant, could make democratic deliberation even rarer, or simply prohibitive for all but the richest clients. It is also true that trial juries are likewise small. But unlike them, deliberative forums on health policy are per 3S, in addition to being small, irregular. That keeps any civic education from these forums a drop in the ocean of the population at large. It is conceivable that the existence of forums of one's peers in the background boosts the trust of nonparticipants even absent special media campaigns, say, through regular mentions in civics classes. The existence and magnitude of that effect is an open empirical question. And for any documented positive effect on public trust the question will still arise whether it emanates entirely from enhanced trustworthiness, or also from manipulation; for example, although 3S largely prevents deliberative forums from serving public will, these forums may boost public trust thanks in part to public obliviousness to this implication of 3S.

While the number and frequency of feasible deliberative forums on health policy is too small to significantly educate the populace, mini‐publics can engage and educate a critical mass of policy makers and experts. For example, they may force policymakers to introduce only policy that lay deliberators are expected to approve. That could force consideration for important details that policymakers chronically neglect despite their importance to users, such as effectiveness on the ground. How much repeated deliberative events on health policy influence policy makers and experts in these ways, without staving off valuable policies that are hard‐sells, is partly an empirical matter.

### 3.9 Equal Respect

Deliberative democracy on health policy is often recommended for its alleged respectfulness toward citizens [e.g., 22, 23, 74]. In particular, showing interest in people's advice, especially on matters of political morality, is thought to reveal appreciation for their moral and intellectual faculties [74]. The deliberative situation itself encapsulates citizens' equal standing by selecting deliberators who are social equals or with whose demographics one identifies (compare to being tried by “a jury of one's peers”)^32^ and then engaging all as free and equal in the deliberations [8, 26, 30]. Finally, giving them reasons treats them as “in charge”.

Earlier I mentioned that when democratic deliberation increases the public's trust and civic virtue and reduces populist voting, that may just mean that the existence of deliberative forums has misled the public. Democratic deliberation is designed to give citizens the impression that they are listened to. But that impression is often false. 3S makes being genuinely listened to in a deliberative democracy a statistically rare event. Additional aspects of deliberative democratic best practice may likewise be suboptimally respectful of the lay public. Forum organizers may express great interest in what a delegate invited *qua* member of a certain demographic group says “as” a member, despite understanding that, given 3S, the next member of that demographic might have thoroughly different opinions.

There is a further problem with the equal‐respect defense. In Kantian and democratic‐egalitarian traditions that demand equal respect, our basic status as citizens is considered equal, not near‐equal, and not somewhat more equal than under alternative arrangements. But on the equal‐respect defense's seeming implicit assumption, that respect for a citizen's value or status hinges on her level of participation in deliberative forums, 3S's implication, that only a minority of the populace takes part in those forums, would mean that a deliberative democracy treats citizens as unequal. In the foreseeable future, only some of them will participate in deliberative forums, per 3S. If alternatively the level of respect for a citizen hinges on her voice and influence in political life in general [83], professional experts and officials are paid more respect than lay delegates so long as experts and officials maintain some added role in decisionmaking: only the most radical lottocrats would do away with experts and officials altogether (Landemore writes that she would not). Intensive participation for some and, per 3S, only sparse participation for others, does not express that they are equals. It actively expresses that they are unequal. Either way, the message of democratic deliberation on that implicit assumption is that we are unequal.

A possible response is that all lay members of the public, including ones not randomized for active participation, are amply represented by those lay members randomized for active participation. Because delegates are respected as equals in the forum, all citizens are shown equal respect through each deliberative forum, rare as it may be for a citizen to participate. But if so, why doesn't classical representative government do the same, and provides equal respect to all citizens—through everyone's official representatives? Democratic deliberation is suposed to be an *alternative* to representative government.

Perhaps what democratic deliberation achieves in terms of (equal) respect is giving all citizens the *soothing sense* that they have been treated with (equal) respect, thereby helpfully elevating their self‐esteem, trust, and cooperation, or perhaps even their sense of self‐respect? That soothing effect may be imagined to affect deliberators alone, or to everyone. There is a problem in the argument either way. If the soothing effect accrues to deliberators then, per 3S, deliberations' pacifying impact on society at large remains negligible, and hardly worth the cost. If alternatively deliberations calm all citizens, say, by giving all a reassuring sense that they have been treated with equal respect although the existence of deliberative forums did *not* give them equal respect, then everyone's improved feeling about themselves seems to be based on manipulation, using their failure to appreciate how limited the equal respect on offer was.

We have seen that 3S poses problems for the prevalent arguments for best‐practiced democratic deliberation on nonlocal health policy. Remember that many of the alleged merits of democratic deliberation on health policy and in general have been said to make democratic deliberation important, specifically, to legitimacy. It is ironic that the modest merits we were able to confirm in democratic deliberation despite 3S are not lofty matters of basic political legitimacy. These merits were more pragmatic and contingent: giving the public the—potentially false—impression that it has been thoroughly consulted and can trust resulting decisions. The potential illusion that everyone has been taken seriously. *Modus vivendi*. Collecting input to discover widespread confusions early. An occasional good idea.

## 4 Conclusion

The 3S challenge weakens most contemporary arguments for proper democratic deliberation on regional, national and global health policy under current conditions. It especially questions dramatic visions of democratic deliberation as an alleged key to political legitimacy, and revelatory of the people's binding will.

A full assessment of the normative upshots of 3S for inclusive deliberation on health policy will depend on vetting the myriad supportive arguments for such deliberation under various feasible scenarios. Best‐practice democratic deliberation is only one element of the best mosaic of health policy design methods. Like referenda, representative government, health‐state utility surveys, and expert decisionmaking, it is functional sometimes and a bad idea on others. Variants on classical democratic deliberation that part ways with this or that component thereof could also prove helpful sometimes. Depending on circumstances, it might be smarter on occasion to collect only qualitative input without quantitative polling; to avoid any informing or deliberating which may bias qualitative findings; to engage experts when they would inspire greater trust or reach urgent decisions faster than nonexperts; or, on those circumstances that descriptive representation is less functional [84], to forego inclusivity altogether. What is unlikely is that democratic deliberation or one form thereof will be found a one‐size‐fits‐all key to legitimacy, to continued public trust, and to other crucial moral requirements.

While my focus was health policy, I suspect that both the 3S challenge to proper democratic deliberation and this mosaic picture of proper policy design apply outside health policy as well. If so, 3S would challenge the deliberative democratic project more broadly.

## Conflicts of Interest

The author declares no conflicts of interest.

## Data Availability

The author has nothing to report.

## Endnotes

^1^
For classical and recent definitions of democratic deliberation, see [4, 5, 6, 7, 8, 9]. For definitions of democratic deliberation on health policy, see [10, 11, 12, 13, 14, 15, 16].

^2^
Interestingly, inclusivity is absent from some early definitions of deliberative democracy, for example, “decision making by discussion among free and equal citizens” [25, and see likewise 13, 26]. These failures to mention inclusivity may have been unintended; some of the same authors elsewhere demand inclusivity, for example, “an inclusive approach to stakeholder involvement is always preferable” [12].

^3^
See for example, “In addition to focusing on individual knowledge, preference, and voting, deliberative democracy also incorporates inclusive participation that encompasses citizens and leaders, mutual justification, listening, respect, reflection, and openness to persuasion” [1, 18].

^4^
Accordingly, political and legislative impact to deliberation usually excites deliberative democrats [1] and its persistent absence frustrates them [27].

^5^
Reaching consensus is no longer considered crucial [1, 26].

^6^
An alternative account of the role of “legitimacy” in defenses of democratic deliberation is that it merely adds detail to the case for democratic deliberation, without bolstering it. In that vein, if justice issues demands but legitimacy issues only constraints, to say that democratic deliberation is called for as a matter of legitimacy may merely reveal the negative nature of democratic deliberation's normative upshots—for example, that while there is no duty to deliberate, absent deliberation all policy measures are forbidden. This alternative account of “legitimacy” can still make democratic deliberation absolutely essential, morally.

^7^
Seeking consensus in democratic deliberation would further increase per‐participant costs [38], but this article sets that largely‐forgone best practice aside.

^8^
It is true that expert and political representative deliberations pay *their* participants much more per hour than democratic deliberation typically pays members of the ordinary public. But the former are typically paid anyhow, whether or not they deliberate. And 3S's basic point is not comparative. Rather, it is that because democratic deliberation is costly per participant then (whether or not the alternative costs even more per participant), the number of participants in democratic deliberation will feasibly remain low.

^9^
Still, one can never exclude the possibility of radically different future circumstances or technologies that would make meaningful dialogue far more scalable. In particular, increasing penetration of video conferencing and communication platforms may make nearly everyone proficient at deliberating meaningfully online. Engaging tiny sectors who still lack online access, as in one Brazilian largely‐online platform that engaged indigenous communities in‐person [43], may remain affordable.

^10^
The 1500 were approached, those who could not speak or read Hebrew were excluded, and the rest were interviewed, with 44% of interviewees expressing interest in participating. Out of those, the actual participants were “selected” [47 does not explain how].

^11^
Admittedly, some deliberative processes with only days of active deliberation include multiple pulses of active deliberation, between which participants return to their ordinary lives and ideas “percolate” for longer periods [47].

^12^
By contrast, taking part in participatory democratic activities [49] can happen frequently and thus *cumulatively* permit all citizens to contribute substantially.

^13^
Admittedly, deliberation can remove one source of noise in nondeliberative surveys: “when being interviewed for surveys people may feel obliged to express off‐the top‐of‐the‐head opinions… on matters they have not had the opportunity to think through” [53]. But that is only one source of random noise among many.

^14^
On one interpretation, Daniels recommends democratic deliberation only for those questions on which there is no consensus *among experts*. Conceivably, he accepts that the general public may be in consensus so overwhelming that even a small poll would detect the majority.

^15^
The size of deliberative forums tends to remain too small whether we use the effect size of minimum practical significance or the smallest effect size that begins to have practical relevance.

^16^
Reports on Deliberative Polling® do not always stick to that official focus. In a report on a recent global survey on personal use of AI, the only results explicitly stated to meet a 95% or greater confidence interval concerned changes in opinion before/after the deliberation. Yet the report called attention to other results as well because “considered opinions are also noteworthy” [54]. In many Deliberative Polls®, the per cent of the public and of particular sectors who selected this or that answer (not just changes therein) are featured, with no perspicuous warning to readers and TV viewers to disregard these numbers [55].

^17^
As in recent Deliberative Polling® for a deep‐pocketed corporation with ready access to computer‐savvy online users [54].

^18^
A 24‐year old African American male from the north might furthermore not see himself in a 64‐year old African‐American male from the south. So any positive effects (e.g. on trust and on the sense that one is being treated with respect—see subsections 3.8 and 3.9 below) coming from the recruitment of people he sees himself in might be nil if the forum is too small to include members in his demographic, understood narrowly—in this case, to reflect also US region‐ and age group.

^19^
I thank Bill Hallman for this point. There are additional systematic biases in democratic deliberation, such as social desirability bias and the tendencies of self‐confident (often privileged) delegates to dominate discussions and of the experts running information sessions to frame discussions. Smart design can reduce some of those. My focus is on the biases emanating from 3S.

^20^
One might think that in countries without democratic elections or representative‐accountability, even suboptimal democratic deliberation could only improve the representation of public will [for examples of democratic deliberation on health policy in undemocratic societies, see 57, n. 18]. Nevertheless, many undemocratic countries are poor, making the humanitarian opportunity‐cost of expensive deliberative forums utterly prohibitive (see Leah Pierson's contribution to this symposium).

^21^
An underlying assumption is that the right policy decisions depend among other things on normative truths. I am not questioning that assumption (or making it), only the advantage of democratic deliberation in tracking those truths if any exist.

^22^
See also Fishkin's admission, “If it is a question of expertise, surely professional experts can be found whose “advice” might be more valuable because of their technical training or proficiency” [52].

^23^
In addition, the “Diversity Trumps Ability” Theorem is considered by some to rest on “essential and irreparable errors” [68]. I am grateful to an annonymous reviewer for Bioethics for pointing that out.

^24^
For some purposes, therefore, uninformed democratic deliberation could prove more useful than current best practice.

^25^
Admittedly, unlike most essay prize decisions, health policy decisions should typically be “sellable” to the lay public, which having lay decision makers may facilitate. But “sellable” is not what Landemore means by the “best” policy.

^26^
In addition, as an anonymous reviewer for *Bioethics* pointed out to me, “Condorcet's Theorem would not clearly apply, insofar as it requires judges to be probabilistically independent and the point of deliberation is that people persuade each other.” The reviewer also conceded that there are recent attempts to relax the independence requirement for democratic deliberation.

^27^
According to legend, Colonel William Travis, commander of the defense forces of the Alamo fortress, realized that continuing defense would result in defenders' death. With his sword, he drew a line in the sand. The death mission included only soldiers who stepped over that line—a (re‐)consent.

^28^
This argument for democratic deliberation assumes that more egalitarian or more prioritarian policy is fairer. That assumption may contradict the motivating thought [31] that precisely how much our policies should in fairness be egalitarian or prioritarian is itself reasonably contested.

^29^
But those things are not always good for us. Consider Disney heiress Abigail Disney, who holds that ethically she should be taxed more. Would it benefit her to influence taxation policy—or disadvantage her, since it might increase her taxes? Additionally, riffing on Oscar Wilde, one might quip: *The trouble with democratic deliberation is that it takes up too many evenings*; Americans, at least, may prefer to keep evenings free [67]. However, my argument does *not* assume that having voice, deliberating, and influencing can disadvantage us.

^30^
In one way, deliberative democracy boosts fair opportunity to deliberate even less than a tiny hole in a job market's crystal ceiling. The few who make it through such a hole and land a job often especially merit it, providing a modicum of justification. Selection to participate in a deliberative forum is random (element “b” above), reflecting no distinctive ground for the entitlement.

^31^
Such claims are often hard to assess because many supporters of deliberative democracy vacillate between descriptive and normative senses of legitimacy—some defenses allege stronger trust post‐deliberation and others, stronger reasons for trust post‐deliberation. Even if the normative case for democratic deliberation is valid, expert decision making might preserve actual trust more. Experts are trained among other things to put their conclusions in a “narrative that inspires confidence” [50]. The inclusion of underrepresented minorities (element “a” above) may weaken a racist general public's trust—and there are indications that that could affect overall trust more than any gains in trust among formerly‐underrepresented minorities, and despite the reasons for trust that it creates: US voters’ trust of congress is more responsive to their racial concordance with their congressional representative among whites than among blacks [82]. Deliberation supporters chronically fudge the crucial distinction between descriptive and normative legitimacy. Even philosophically‐sophisticated supporters have recently cited normative thinker Rawls as somehow an authority that deliberation facilitates legitimacy undestood descriptively [23]; they distinguish descriptive from normative legitimacy only in later work in progress [46].

^32^
I thank an anonymous *Bioethics* reviewer for pointing that out to me.
